# Fermented Fish Products: Balancing Tradition and Innovation for Improved Quality

**DOI:** 10.3390/foods13162565

**Published:** 2024-08-16

**Authors:** Hang Li, Guantian Li, Yunchen Bi, Song Liu

**Affiliations:** 1 CAS and Shandong Province Key Laboratory of Experimental Marine Biology, Center for Ocean Mega-Science, Institute of Oceanology, Chinese Academy of Sciences, 7 Nanhai Road, Qingdao 266071, Chinayunchenbi@qdio.ac.cn (Y.B.); 2 Laboratory for Marine Biology and Biotechnology, Qingdao Marine Science and Technology Center, Qingdao 266237, China; 3 Laboratory for Marine Drugs and Bioproducts, Qingdao Marine Science and Technology Center, Qingdao 266237, China

**Keywords:** microbial metabolism, traditional food, salted fish, fish sauce, ethnic food

## Abstract

The flavor profile of fermented fish products is influenced by the complex interplay of microbial and enzymatic actions on the raw materials. This review summarizes the various factors contributing to the unique taste and aroma of these traditional foods. Key ingredients include locally sourced fish species and a variety of spices and seasonings that enhance flavor while serving as cultural markers. Starter cultures also play a critical role in standardizing quality and accelerating fermentation. Flavor compounds in fermented fish are primarily derived from the metabolism of carbohydrates, lipids, and proteins, producing a diverse array of free amino acids, peptides, and volatile compounds such as aldehydes, ketones, alcohols, and esters. The fermentation process can be shortened by certain methods to reduce production time and costs, allowing for faster product turnover and increased profitability in the fermented fish market. Fermented fish products also show potent beneficial effects. This review highlights the importance of integrating traditional practices with modern scientific approaches. Future research directions to enhance the quality of fermented fish products are suggested.

## 1. Introduction

Fermented fish products hold a significant place in the culinary traditions of various cultures worldwide [1]. These products, resulting from the controlled microbial breakdown of fish, offer a unique blend of flavors, aromas, and textures that have captivated palates for centuries. Examples of fermented whole fish or fish pieces products include *adjuevan* in Côte d’Ivoire [2], *ayu-narezushi* in Japan [3], *chouguiyu* and *suanyu* in China [4,5], *hákarl* in Iceland [6], *pha-ork kontrey* in Cambodia [7], *pla-duk-ra* and *pla-khem-neur-som* in Thailand [8,9], *rakfisk* in Norway [10], and *surströmming* in Sweden [11]. In addition to the solid form of fermented fish products, fish can be processed into fish sauce, a seasoning liquid usually with a red-amber appearance, such as *budu* in Malaysia [12], *colatura di alici* in Italy [13], *ishiri* in Japan [14], *mahyaveh* in Iran [15], and *yulu* in China [16]. Traditionally, it may take more than one year for the liquefaction of fish muscle to produce fish sauce [17]. Fermented fish can also be processed into pastes, such as *padaek* in Laos [18]. Fermented fish pastes are mainly consumed as condiments for flavor enhancement [18,19]. Beyond their culinary appeal, fermented fish products also boast a rich history, cultural significance, and potential health benefits. The fermentation process not only enhances the sensory attributes of fish but also acts as a natural preservation method, extending its shelf-life and making it a valuable source of protein and nutrients in many regions [2,3,6,7,9,10,11].

Traditionally, the fermentation process is initialized by the autochthonous microorganisms naturally existing on the fish and other raw ingredients as well as the surrounding environment [20]. Traditional fermentation methods often vary widely between regions, communities, and even individual producers, leading to inconsistencies in the quality and safety of the final products [2,12,13,21]. These variations pose significant challenges in establishing and maintaining uniform quality control measures, which are essential for ensuring consumer safety and satisfaction. The inoculation of starter cultures offers a promising approach to address these issues by allowing the dominant growth of desired strains from the early stage of the fermentation. The propagation of lactic acid bacteria (LAB) can produce acid and thus lower the pH, which can inhibit the growth of undesired strains [7]. The antimicrobial activity of the bacteriocins produced by certain LAB strains can also contribute to the inhibition of spoilage strains [22]. In addition to LAB, microorganisms including archaea [23], yeast [24], and mold [25] can also be used as starter cultures. These microorganisms contribute to the diversity of flavor compounds in fermented fish products.

During fermentation, the carbohydrates, lipids, and proteins in raw fish are degraded into flavor compounds (e.g., glutamic acid and aspartic acid) and volatiles (e.g., aldehydes, alcohols, and ketones) through the action of indigenous and microbial enzymes [26]. The possible metabolic pathways include Ehrlich pathway, Harris pathway, and Strecker pathway [27,28]. Flavor-presenting free amino acids (e.g., glutamic acid) [19] and peptides (e.g., umami-tasting peptide DEEYPDLS) [29] are also generated, owing to the degradation of proteins. The introduction of spices (e.g., aniseed) can further enrich the aromatic profiles of the final products [4,19,30]. However, unfavorable volatiles (e.g., sulfur- and nitrogen-containing compounds) that contribute to unpleasant odor may also be present in fermented fish products [5,31]. Developing strategies to manage the flavor profile of fermented fish products remains a critical area of research that requires further investigation.

There is also a growing interest in developing strategies to improve their production, preservation, and health benefits. Accelerating the fermentation process is one of the key challenges faced by the industry, as traditional methods can be time consuming and may lead to inconsistencies in product quality. Recent studies have explored the use of starter cultures [32], exogenous enzymes [33], and novel processing technologies [34] to shorten fermentation time while maintaining the desired sensory attributes. Moreover, there is an increasing focus on understanding the potential biological effects of fermented fish products, as they contain a wide range of bioactive compounds that may offer health benefits beyond basic nutrition. Studies have suggested that these products may possess antioxidant [35], anti-diabetic [36], and gut microbiota-modulating properties [25], among others. However, further research is needed to validate these findings and elucidate the underlying mechanisms of action.

Focusing on recent publications, this review summarizes recent developments of fermented products with emphasis on the ingredients and flavor compounds. The relation between flavor compounds and core microorganisms are discussed. Methods to speed up the fermentation process are introduced. Potent health benefits of fermented fish products are also summarized in this review.

## 2. Ingredients Used for Fish Fermentation

### 2.1. Raw Materials for Fermentation

The raw materials for fish fermentation, particularly the fish itself, are usually locally sourced and readily available. Traditionally, the choice of fish species depends on the geographical location and the abundance of specific fish in the region. For example, grass carp (*Ctenopharyngodon idellus*) [25,37], common carp (*Cyprinus carpio* L.) [26,38], and mandarin fish (*Siniperca chuatsi*) [31,39], species abundant in China, have been frequently used in Chinese fermented fish products. This approach not only ensures the freshness of the fish but also reflects the unique terroir and cultural identity of the fermented fish products. For the production of fish pastes and sauces, the transformation of fish from solid state to semi-solid or liquid state is a key process, which means that maintaining the textural integrity of the fish muscle is not a primary concern. This allows for the use of a wide range of raw materials, including low-value fish and side-stream products such as fish heads [40], viscera [41], and bones [24]. Utilizing these undervalued resources provides economic benefits while promoting environmental sustainability by reducing waste. The flexibility in raw material selection for fermented fish production opens up opportunities for valorizing underutilized fish species and byproducts.

### 2.2. Seasoning Ingredients for Fermented Fish Products

The use of seasoning ingredients such as salt and spices plays an important role in the production of fermented fish products. Salt is the most essential seasoning ingredient, as it not only enhances the flavor but also acts as a preservative by inhibiting the growth of spoilage and pathogenic microorganisms while promoting the growth of halophilic microorganisms [42,43]. In addition to salt, various spices are commonly used in fermented fish. The role of spices in fermented fish products is understood not only from a flavoring perspective but also serves as a cultural marker, distinguishing the fermented fish products of one region from another [44,45]. For example, the use of chili and galangal in the production of Thai fermented fish products imparts a distinctive spicy and aromatic flavor that is characteristic of Thai cuisine [8,9]. Similarly, the use of aniseed, cinnamon, and fennel in Chinese fermented fish products such as *suanyu* and *chouguiyu* contributes to their unique flavor profiles and cultural identity [4,19,30]. Spices not only enhance the sensory quality of fermented fish products but also may help to mask undesirable odors and flavors that may arise during the fermentation process [5,46]. The complex mixture of volatile compounds present in spices, such as terpenes, phenols, and esters, interact with the flavor compounds produced by the fish and microorganisms, creating a synergistic effect that results in the distinctive aroma and taste of fermented fish products [19,30]. As the demand for these products grows, there is a need for further research to optimize the use of seasoning ingredients and balance their functional properties with the changing preferences of consumers and the need for healthier and more sustainable food products.

### 2.3. Starter Cultures for Fermented Fish Products

The fermentation of fish can be initiated by inoculating starter cultures, which helps to standardize the quality characteristics of the final product. Various starter cultures, including bacteria, archaea, yeast, and mold, have been used in recent studies for fish fermentation (Table 1). These starter cultures can be obtained commercially or isolated from the microbial succession during the fermentation process. Traditionally, fermented fish products have a relatively high salinity to inhibit the growth of spoilage and pathogenic microorganisms [15,47,48]. As a result, starter cultures used in these products should be tolerant of high salt concentrations. The use of halophilic strains is particularly advantageous, as they can survive and grow rapidly in the extreme environment of salted fish, occupying the empty niches and preventing the colonization of undesired opportunistic microorganisms.

#### 2.3.1. Bacteria and Archaea as Starter Cultures

LAB strains are ideal starter cultures for fish fermentation. Indeed, LAB genera are the dominant microbial genera and highly conducive to the formation of flavor compounds in many fermented fish products [5,28,60]. LAB strains can acidify the environment and suppress the growth of undesired microorganisms. For example, *Lactiplantibacillus plantarum* CP-134 (GenBank ID: MK601693.1), a salt-tolerant (20% NaCl) strain, was shown to rapidly produce acid to decrease pH to 3.6 in three days [60]. Certain LAB strains also exhibit antimicrobial activity, likely owing to the substances they secreted, such as hydrogen peroxide and bacteriocin [61]. Selection of LAB strains with antimicrobial properties can be an effective strategy for controlling the growth of spoilage bacteria in fermented fish products. For example, *Lactiplantibacillus plantarum* CP-134shows antimicrobial activity against *Staphylococcus aureus* and *Escherichia coli* [60]. Similarly, *Lactiplantibacillus plantarum* CGMCC 20032 shows inhibitory effects on *Staphylococcus aureus*, *Aeromonas hydrophila*, *Aeromonas veronii*, *Cronobacter sakazakii*, and *Salmonella* Typhimurium [51]. In addition to their antimicrobial activity, LAB strains (*Lactobacillus acidophilus*, *Lactobacillus bulgaricus*, and *Lacticaseibacillus casei*) are also capable of removing aflatoxin B_1_ and T-2 toxin from fermented fish products [62]. Furthermore, LAB strains can produce gamma-aminobutyric acid (GABA), the substance that exhibits multiple functions [63]. In some studies, high-GABA-producing strains (*Pedioccocus pentosaceus* and *Lactiplantibacillus pentosus*) were screened from different fermented fish products [64,65]. These strains could potentially be used in the production of GABA-enriched fermented fish products, offering additional health benefits to consumers.

Archaea also play a significant role as starter cultures in fish fermentation. For example, the proportion of archaea in different Indian fermented fish products might be up to 87.6% of the total halophilic microbial communities, suggesting their importance in flavor development [66]. Screened archaeal strains show rapid growth rates and high protease and lipase activities, which are essential for the breakdown of fish proteins and lipids during fermentation [32]. Additionally, the propagation of archaea also contributes to the reduction of pH though the production of organic acids [32]. The use of halophilic archaea as starter cultures required high salinity. For example, the optimum NaCl concentration for five strains of *Halobacterium salinarum* DSM 3754^T^ varied from 20% to 30% [23]. The use of bacteria and archaea as starter cultures presents an opportunity to develop unique and flavorful fermented fish products, especially in high-salt environments. However, further research is needed to understand the specific contributions of bacteria and archaeal strains to the sensory profiles and safety of fermented fish products, as well as to optimize their use in commercial fermentation processes.

#### 2.3.2. Fungi as Starter Cultures

In addition to bacterial and archaeal starter cultures, fungi, including yeasts and molds, have been employed in the fermentation of fish products [19,25,50]. The inoculation of yeasts, such as *Pichia anomala* and *Saccharomyces cerevisiae*, has been shown to enhance the production of volatile compounds in final products [19,24,50]. Moreover, the co-inoculation of yeast and LAB strains may synergistically improve the sensory quality of fermented fish products. For example, the co-inoculation of *Saccharomyces cerevisiae* 22 and LAB strains (*Pediococcus pentosaceus* 220 and *Staphylococcus xylosus* 135) in grass carp resulted in higher levels of favorable free peptides and volatiles, as well as improved sensory scores, compared to the individual inoculation of yeast or LAB strains [24]. Similar results were observed in the co-inoculation of *Lactiplantibacillus plantarum* and *Saccharomyces cerevisiae* on fermented grass carp [50]. The combination of *Pichia anomala* with different LAB strains (*Pediococcus acidilactici*, *Pediococcus pentosaceus*, *Staphylococcus carnosus*, and *Staphylococcus xylosus*) has also been reported to produce fish–chili pastes with different sensory characteristics [19]. Mold starter cultures have also been explored in fish fermentation. The inoculation of *Monascus purpureus* Went M 3.439 in the form of red yeast rice promoted the in vitro digestibility of fish protein [25]. *Aspergillus oryzae* S. NPUST-FS-206-A1, in the form of black bean koji, has been used for fish fermentation, with an optimal pH of 5 for its protease activity [59]. The use of fungal starter cultures in fish fermentation offers several advantages including the production of unique flavor compounds and the potential for synergistic effects when combined with bacterial starter cultures [19,67]. However, further research is needed to optimize the use of fungal starter cultures in fish fermentation, particularly in terms of strain selection and fermentation conditions. Additionally, the safety aspects of using fungal starter cultures should be carefully considered, as some fungal species may produce mycotoxins or other harmful compounds [68,69].

### 2.4. Addition of Exogeneous Enzymes

The direct addition of enzymes is an emerging strategy to improve the quality and accelerate the fermentation process of fermented fish products. For example, the application of papain to spontaneously fermented Chinese perch promotes the release of free amino acids, volatiles, eicosapentaenoi acid (20:5 n-3), and docosapentenoic acid (22:5 n-6) [70]. Interestingly, the addition of papain also significantly increases the relative abundance of Lactobacillales, which are positively correlated with volatiles [70]. The application of the proteases from *Aspergillus oryzae* in the form of Flavourzyme^®^ led to lower water activity among fermented grass carp, probably due to the further weakening of the water holding capacity induced by the increased protein hydrolysis [53]. The addition of Flavourzyme^®^ accelerated the acidification during the early fermentation stage, but it had little effect on the final pH of the product [53]. The addition of lipase to fermented carp significantly enriches the volatile compound without significantly altering the pH [33,71]. The abundance of *Proteus*, a genus known for its lipolytic activity, is also positively correlated with the production of volatile compounds in lipase-treated fermented carp [33]. Other exogenous enzymes (e.g., alcalase, bacillolysin, bromelain, ficin, pepsin, subtilisin, and trypsin) [72], which may facilitate hydrolysis, may also be potential candidates for application in fish fermentation. However, further research is needed to evaluate their effects on the quality, safety, and sensory properties of fermented fish products. The potential synergistic effects of combining exogenous enzymes with starter cultures should be explored to maximize the benefits of both approaches.

### 2.5. General Discussion on Ingredients Used for Fish Fermentation

The selection of raw materials, seasoning ingredients, starter cultures, and exogenous enzymes is crucial for determining the quality, safety, and sensory properties of fermented fish products. However, several aspects of the current research and practices in this field require further investigation and improvement. The influence of salt source on the microbial community and sensory properties of fermented fish products has not been extensively studied. The diversity of microorganisms associated with different salt sources can contribute to unique characteristics of fermented fish products from different regions. A recent study found that marine-sourced salt products were dominated by archaea (e.g., *Halorubrum*, *Halobacterium*, and *Hallobellus*), while other salt products (Himalayan pink, Hawaiian black, and Viking salt) primarily harbored bacteria (e.g., *Sulfitobacter* sp., *Bacillus*, and *Enterococcus*) [73]. The impact of these salt-associated microorganisms on the fermentation process and the final product quality remains to be elucidated. The use of starter cultures, although ensuring consistent quality, may not fully capture the complex interactions between different strains in mixed starter cultures and the activity of exogenous enzymes throughout the fermentation process. The studies discussed in this review provide valuable insights, but they may not have adequately addressed the potential synergistic or antagonistic effects of these interactions on the final product quality. Moreover, the reliance on strains isolated from qualified products (e.g., microbial succession from previously developed products) as an alternative approach to ensure desired characteristics may limit the exploration of novel and potentially beneficial microorganisms. Researchers should also consider the genetic stability and adaptability of these isolated strains to various fermentation conditions and raw materials.

While traditional methods may offer insights into culturally preferred flavors and techniques, they may not always align with modern food safety standards, quality control measures, and scientific understanding of the fermentation process [74,75,76]. Traditional fermentation methods may rely on uncontrolled or spontaneous fermentation, which can allow the growth of harmful bacteria alongside the desired microorganisms [6,42]. In addition, traditional methods may not take into account the complex microbial interactions, biochemical processes, and environmental factors that influence the quality and safety of the final product [17,77]. This lack of scientific understanding can limit the ability to optimize the fermentation process, control potential risks, and develop new and improved products. Therefore, the development of fermented fish products should prioritize the use of starter cultures, exogenous enzymes, and other scientifically validated ingredients and techniques. These modern approaches allow for greater control over the fermentation process, reducing the risk of contamination and ensuring the production of safe and high-quality products [78,79,80,81].

## 3. Flavor Compounds in Fermented Fish Products

The formation of flavor compounds in fermented fish products is a complex process that involves the metabolism of carbohydrates, lipids, and proteins under the action of microbial and endogenous enzymes [2,28,30,82]. The specific flavor profile of a fermented fish product is influenced by various factors, including the microbial community and fermentation conditions. This section provides an overview of the main taste-presenting and aromatic compounds, as well as their possible precursors and the microorganisms involved in their formation. The development of genome-sequencing technologies, such as next-generation sequencing, has accelerated the exploitation of microbial profiles of fermented fish products [83]. Meta-omics approaches, including metagenomics and metatranscriptomics, can effectively highlight the correlation between flavor compounds and core microorganisms [31,56,84]. By identifying the key microorganisms and their metabolic pathways involved in the production of desirable flavor compounds, researchers can gain valuable insights into the complex biochemical processes that shape the unique sensory profiles of fermented fish products. This knowledge can be applied to optimize fermentation conditions, select appropriate starter cultures, and manipulate the availability of flavor precursors to enhance the organoleptic properties of these traditional foods. Furthermore, understanding the microbial ecology of fermented fish products can inform strategies to minimize the formation of off-flavors and ensure consistent product quality [85]. Therefore, this section also discusses the microorganisms related to the flavor compounds, emphasizing the importance of controlling flavor precursors and related microorganisms to improve the organoleptic properties of fermented fish products.

### 3.1. Free Amino Acids and Peptides

Free amino acids and peptides, derived from the proteolysis of fish proteins, are major contributors to the flavor profile of fermented fish products [26]. Umami-tasting free amino acids such as glutamic acid and aspartic acid, and sweet-tasting amino acids such as alanine, glycine, proline, serine, and threonine can impart a favorable taste to the final product [19]. However, proteolysis can also lead to the formation of bitter-tasting amino acids such as arginine, histidine, isoleucine, leucine, methionine, phenylalanine, tyrosine, and valine [5,37]. The balance between favorable and bitter-tasting amino acids can be influenced by the action of endogenous and microbial proteases during fermentation [5,40]. In a study on the peptidase genes in raw grass carp, MER0427791 was identified as the predominant endogenous peptidase gene [37]. In fermented mandarin fish, the most abundant umami peptides were ADKEIEDLK, EEVGVEEEKP, GEKVDFD, KVDFDDIQK, TVETEKTEIQ, and VDFDDIQK, which were primarily derived from troponin and myosin [86]. Among six peptides derived from fermented sea bass, peptide DEEYPDLS had the most intensive umami flavor, followed by DEEYPDL, EEEVVEEVE, DEGDLDF, DGEKVDFDD, and EPEPEPEPE [29].

The inoculation of LAB can promote the proteolysis of fish muscle. *Stenotrophomonas* and *Tetragenococcus* have been significantly positively correlated with umami amino acids (e.g., glutamic acid and aspartic acid) and sweet amino acids (alanine, threonine, serine, glycine, and proline) of fish sauce [16]. In fermented mandarin fish, six core umami peptides were significantly correlated with the LAB genera *Enterococcus*, *Lactococcus*, *Peptostreptococcus*, *Streptococcus*, and *Vagococcus* [86]. The mackerel inoculated with *Latilactobacillus sakei* had a higher level of umami amino acids and sweet-tasting amino acids than those inoculated with *Lactiplantibacillus plantarum* or *Weissella cibaria* [57]. Non-LAB genera, such as *Acinetobacter*, *Arcobacter*, *Fusobacterium*, *Oceanisphaera*, and *Psychrilyobacter*, have also shown correlations with taste-related peptides [5,86]. *Macrococcus* and *Staphylococcus* are more closely related to the production of free amino acids compared to other non-LAB strains [37]. The flavor-enhancing dipeptides (e.g., glutamylleucine) were also positively correlated with the abundance of *Aerococcus*, *Cobetia*, *Halomonas*, and *Psychrobacter* [87]. The level of bitter-flavored dipeptide leucylproline could be enhanced by the growth of *Staphylococcus* and *Cobetia* [87].

The generation of flavor amino acids and peptides is dependent on microbial proteases. In fermented grass carp, for example, approximately 40 out of the top 50 peptidase genes were annotated to the genus *Macrococcus* [37]. However, the proteinase activity of LAB is strain specific. A previous study found that only eight LAB strains (GenBank ID: MG383778.1, MG754574.1, LC379973.1, KX139194.1, MK156350.1, MF423845.1, AB368912.1, and JQ043368.1) out of 42 dominant LAB strains isolated from *suanyu*, a traditional Chinese fermented fish product, showed proteinase activities [60]. Microbial aspartic endopeptidases were found to be associated with the genera *Acinetobacter*, *Vagococcus*, *Psychrobacter, Myroides*, *Citrobacter*, and *Hafnia* [86]. Cysteine endopeptidases were linked to the genera *Aeromonas*, *Citrobacter*, *Kluyvera*, *Pseudoalteromonas*, *Pseudomonas*, *Psychrobacter*, and *Vibrio* [86]. Serine endopeptidases can be found in LAB of *Enterococcus*, *Lactococcus*, *Peptostreptococcus*, *Streptococcus*, and *Vagococcus*, as well as other genera including *Acinetobacter*, *Carnobacterium*, *Lysobacter*, *Myroides*, and *Psychrobacter* [86]. The bitter-tasting amino acid arginine was related to specific peptidase genes (MER0700839, MER0199362, MER0208403 and MER0207602) [37]. Future studies could employ genome-editing techniques to produce specific strains harboring desirable protease genes for flavor-enhanced fermentation.

### 3.2. Volatiles of Fermented Fish Products

**Figure 1:**
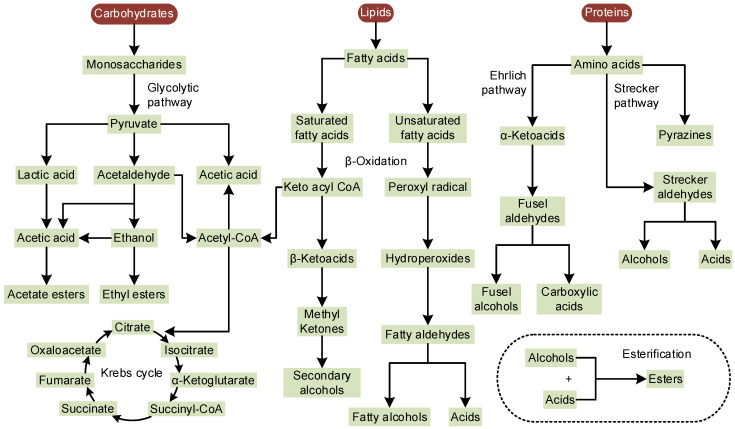
Possible metabolic pathways for the formation of volatiles [31,77,93].

Volatile compounds play a crucial role in the aromatic profiles of fermented fish products, contributing to their distinctive sensory characteristics. These compounds can be analyzed by gas chromatography–ion mobility spectrometry (GC–IMS), gas chromatograph–mass spectrometer (GC–MS), and electronic nose [27,28,31,88,89]. The formation of volatile compounds in fermented fish products is primarily driven by the metabolism of amino acids, fatty acids, and carbohydrates through a complex network of biochemical pathways (Figure 1). The Ehrlich pathway, Harris pathway, and Strecker pathway are the main metabolic routes involved in the generation of volatile compounds [90]. In the Ehrlich pathway, amino acids are transaminated to α-keto acids, followed by decarboxylation to fusel aldehydes and subsequent reduction or oxidation to corresponding fusel alcohols (also known as higher alcohols) or acids [91]. The Harris pathway, or anabolic pathway, involves the production of α-keto acids through the anabolic metabolism of carbohydrates [90]. Strecker degradation, a minor pathway of the Maillard reaction, involves the reaction of α-amino acids and α-dicarbonyl compounds, resulting in the formation of aldehydes through oxidative deamination and decarboxylation [92]. These volatiles greatly contribute to the characteristic flavors of fermented fish products. The representative volatiles found in various fermented fish products are summarized in Table 2.

#### 3.2.1. Aldehydes and Ketones

Aldehydes can significantly impact aroma owing to their generally low odor thresholds [38]. The total abundance of aldehydes typically shows an overall downward trend as fermentation progresses, likely due to their conversion to alcohols and acids [19]. The main characteristic aldehydes in fermented fish products include hexanal (grass, tallow, fat), nonanal (fat, citrus, green), 3-methylthiopropanal (malty, chocolate, caramel), octanal (fat, soap, lemon, green), 2-octenal (roast, fatty), and benzaldehyde (fruity, almond, sweet) [4,28,38,84]. Branched-chain saturated aldehydes (e.g., 2/3-methylbutanal) are usually derived from the Strecker-degraded valine and leucine [28]. Straight-chain aldehydes, including heptanal, hexanal, nonanal, and octanal, are primarily produced from the auto- and enzymatic oxidation of unsaturated fatty acids [28,89]. Oleic acid, which is abundant in fish products, is a major precursor of volatile compounds [89]. It was speculated that oleic acid can be degraded into 10-hydroperoxide, 11-hydroperoxide, and 8-hydroperoxide through different oxidation routes, which were further converted into nonanal, octanal, and decanal, respectively [89]. Hexanal, the metabolite of oxidized linoleic, γ-linolenic, and arachidonic acids, has been reported to increase fourfold during the fermentation of fish [94]. Benzaldehyde, a ubiquitous phenyl aldehyde in fish products with an almond-like flavor, can be generated from Strecker-degraded phenylalanine or oxidative decomposition of linolenic acid [27,28]. *Lactiplantibacillus plantarum* is positively correlated with the production of benzaldehyde [50]. The formation of aldehydes in the early stages of fermentation is dependent on the catalytic activity of lipoxygenase and hydroperoxide lyase, as evidenced by the positive correlation between the accumulation of aldehydes and the activity of these enzymes [26]. The increased activity of lipoxygenase during fermentation may be attributed to the destruction of fish muscle cell membranes, which accelerate the infiltration of salt into muscle cells and elevate lipoxygenase activity [94].

**Table 2:** Representative volatiles and their contents in fermented fish products.

Volatiles	CAS#	Sensory Note	Threshold (μg/kg)	Content (μg/kg)
Aldehydes				
Acetaldehyde	75-07-0	Pungent	15 ^1^	31,545 ^1^
Hexanal	66-25-1	Grass, tallow, fat	5 ^1^	19,597.95 ^2^–56,580 ^1^
Heptanal	111-71-7	Green, rancid	2.8 ^3^	10.09 ^3^–21.3 ^4^
(E)-2-Nonenal	18829-56-6	Green, fatty, tallow	0.19 ^5^	50.13 ^5^–30.9 ^6^
Nonanal	124-19-6	Fat, citrus, green	1 ^1^	372.32 ^2^–6227 ^1^
Octanal	124-13-0	Mushroom, grassy	1 ^1^	173.86 ^2^–2360 ^1^
Benzaldehyde	100-52-7	Fruity, almond, sweet	350 ^1^	41.14 ^7^–9100 ^1^
Decanal	112-31-2	Fruity, orange	2 ^4^	2.93 ^3^–31 ^4^
Ketones				
2,3-Pentanedione	600-14-6	Cream, popcorn	1 ^1^	6300 ^1^
3-Hydroxy-2-butanone	51555-24-9	Cream	800 ^1^	39,040 ^1^
2-Nonanone	821-55-6	Fruity, sweet, waxy	5–200 ^6^	4.79 ^7^–41 ^6^
Alcohols				
1-Octen-3-ol	3391-86-4	Mushroom, grassy	1 ^6^	174.5 ^6^–580.27 ^2^
3-Methyl-1-butanol	123-51-3	Brandy smell	250 ^1^	17.30 ^8^–21,500 ^1^
Linalool	78-70-6	Fruity, floral	6 ^4^	96.9 ^6^–600 ^4^
Esters				
Ethyl acetate	141-78-6	Fruity, floral	5 ^1^	949.75 ^2^–7500 ^1^
Ethyl caproate	123-66-0	Fruity, sweet	1 ^2^	653.12 ^2^
Ethyl heptanoate	106-30-9	Fruity, pineapple	2.2 ^2^	145.97 ^2^
Isoamyl acetate	123-92-2	Fruity, banana	2 ^1^	3200 ^1^
Acids				
Acetic acid	64-19-7	Acidic, sour	22,000 ^1^	1574.4 ^6^–22,000 ^1^
Butanoic acid	107-92-6	Cheesy	240 ^1^	1176 ^1^–2477.09 ^8^
Hexanoic acid	142-62-1	Fatty	3000 ^1^	5100 ^1^
Others				
Indole	120-72-9	Fecal, floral	11 ^5^	30.06 ^8^–4877.31 ^5^
Trimethylamine	75-50-3	Fishy astringency	8 ^5^	358.56 ^5^
Dimethyl disulfide	624-92-0	Sulfur, rotten cabbage	1.1 ^5^	20.65 ^8^–94.85 ^5^
Dimethyl trisulfide	3658-80-8	Sulfur, rotten cabbage	1 ^5^	32.10 ^8^–41.15 ^5^

Notes: ^1^ *Suanyu* made from carp fermented at 25 °C for 4 weeks [38]; ^2^ Carp fermented with mixed starter cultures for six weeks [95]; ^3^ Grass carp spontaneously fermented at 10 °C for 6 days [87]; ^4^ Mandarin fish spontaneously fermented at 20 °C for 7 days [39]; ^5^ Mandarin fish spontaneously fermented at 12 °C for 7 days [31]; ^6^ Carp fermented at 15 °C for 24 days [96]; ^7^ Golden pomfret spontaneously fermented for 20 days [94]; ^8^ Sea bass fermented at 28 °C for 20 days [88].

The formation of ketones is similar to that of aldehydes, primarily by the catabolism of amino acids and unsaturated fatty acids [28]. The α-ketoacids from amino acid metabolic pathway can form corresponding aldehydes and ketones [28]. The abundance of ketones varied with different products and fermentation stages. For example, the most abundant ketones in fermented golden pomfret during fermentation were 3,5-octadien-2-one, and 3-octen-2-one, which are the oxidative products of linolenic acid and linoleic acid, respectively [94]. In contrast, the most abundant ketone in fermented carp was 2,3-pentanedione [38]. 6-Methyl-5-hepten-2-one is an important volatile in fermented fish products [19,28]. However, in a study on fermented golden pomfret, this ketone was only detected after 10 days of fermentation [94]. The presence of 6-methyl-5-hepten-2-one may be attributed to the use of pepper [19], which means that this ketone may be minor or absent in the products without this spice. 2,3-Octenedione was the most abundant ketone in raw common carp, but it gradually diminished with the prolongation of fermentation [38]. The contents of 2-heptanone, 2,5-octanedione, and 3-octanone were significantly higher in carp fermented with *Lactiplantibacillus plantarum* compared to the spontaneously fermented counterpart [50].

#### 3.2.2. Alcohols and Esters

Alcohols are typically the second most abundant volatiles in fermented fish products, accounting for approximately 30% of the total volatiles at the end of fermentation [95]. The inoculation of *Saccharomyces cerevisiae* could greatly promote the generation of alcohols [95]. The main alcohols in fermented fish products include 3-methyl-1-butanol (brandy smell) and 1-octen-3-ol (mushroom, grassy) [28]. Short straight-chain alcohols (e.g., ethanol, 1-propanol, and 1-butanol) may be abundant in fermented fish products, but their contribution to the flavor is often minor owing to their high odor thresholds [88]. On the other hand, the long straight-chain and branched alcohols (e.g., 3-methyl-1-butanol, 1-hexanol, and 1-octen-3-ol) can significantly contribute to the aroma profile due to their low odor threshold values [88]. The content of alcohols is usually highest in the early stage (e.g., 1–4 weeks) of the fermentation [5,19] and is positively associated with the increased activity of alcohol dehydrogenase [26]. Alcohol content shows a downward trend with the prolongation of fermentation, likely due to the formation of esters through the reaction of alcohols with carboxylic acids [19]. 1-Octen-3-ol, one of the primary aroma contributors in fermented fish products, is derived from metabolism of unsaturated fatty acids [28,50,95]. Linoleic acid can be oxidized by lipoxygenase to form 10-hydroperoxide, which is subsequently converted to 1-octen-3-ol [27]. The accumulation of 1-octen-3-ol has been positively correlated with genera including *Acinetobacter*, *Citrobacter*, *Plesiomonas*, *Streptococcus*, *Vagococcus*, and *Vibrio* [39]. The sweet rose-like alcohol phenyl ethanol is the most abundant alcohol in fermented carps at the end of fermentation [50,95]. This compound may be derived from the degradation of aspartic acid and phenylalanine via the Ehrlich pathway [95]. Linalool, an aromatic monoterpene alcohol, is also predominant in some fermented fish products, but its presence may be attributed to the use of spices in the raw materials [4,19,30,96].

Esters, known for their fruity and sweet aroma, can become the most abundant volatiles in fermented fish during the late fermentation stage [19,95]. The main esters found in fermented fish include isoamyl acetate (fruity, banana), ethyl caproate (fruity, sweet), ethyl acetate (fruity, floral), and ethyl heptanoate (fruity, pineapple) [95]. The formation of esters in the early stage of fermentation is promoted by the combined action of acyltransferase and esterase [26]. Notably, the activity of esterase is much higher than that of alcohol acyltransferase during this stage. As fermentation progresses, the activity of esterase sharply decreases, while the activity of alcohol acyltransferase increases [26]. However, the activity of alcohol acyltransferase remains relatively stable in the later stage of fermentation, despite the obvious accumulation of esters. This suggests that the availability of substrate alcohol is a key factor affecting the accumulation of esters [26]. In addition, a previous study found that the ester synthase of *Acinetobacter venetianus* SCSMX-3, isolated from fermented fish, showed high activity at pH range of 5–9, and it exhibited the highest activity at pH 8 [97]. In addition, the favorable temperature for the ester synthase ranged from 20 to 40 °C, with the highest activity found at 35 °C [97].

#### 3.2.3. Other Flavor Compounds

The umami-tasting 5′-nucleotides such as adenosine-5′-monophosphate (AMP) and inosine-5′-monophosphate (IMP) are generated from the decomposition of adenosine triphosphate (ATP) [82]. The main biochemical process involves the dephosphorylation of ATP to adenosine diphosphate (ADP) and AMP [31]. Subsequently, AMP can be converted to IMP by deaminase or to adenosine by nucleosidase. Adenosine and IMP can be further dephosphorylated to adenine and inosine, respectively. Both adenine and inosine can be converted to hypoxanthine, the bitter-tasting compound [31]. The 5′-nucleotides, together with amino acids, synergistically contribute to the umami taste [31,82]. The 5′-nucleotides have been positively correlated with *Cobetia*, *Gillisia*, *Staphylococcus*, and *Steroidobacter* [87].

Nitrogen- and sulfur-containing compounds are responsible for the “stinky” odor of some fermented fish products, such as *chouguiyu* [5,31]. Indole, a nitrogenous compound, presents a strong fecal odor at higher concentrations, but it exerts a pleasant floral aroma when at low concentrations [5]. Therefore, the evaporation of indole induced by cooking may improve the sensory quality of fermented fish. There are two possible reactions involved in the biosynthesis of indole. It can be formed from the hydrolytic β-elimination of l-tryptophan via microbial tryptophanase [98], or from catalysis of indoleglycerol phosphate to glyceraldehyde 3-phosphate and indole [31]. Studies have found that the content of indole is positively correlated with the microbial genera *Acinetobacter*, *Citrobacter*, *Psychrilyobacter*, *Streptococcus*, and *Vibrio* [5,39]. Interestingly, the fermentation of carp with *Monascus purpureus* Went M 3.439 resulted in decreased contents of ammonia and indole during a 24-h in vitro fermentation with human fecal microbiota compared to the non-inoculated samples [25]. However, the mechanism behind the formation of these compounds remains unclear [25]. Trimethylamine, which imparts a fishy and astringent flavor, is a metabolic product of trimethylamine oxide reduction, especially under anaerobic conditions [39]. The level of this compound has been positively correlated with the microbial genera *Plesiomonas*, *Vagococcus*, and *Vibrio* [39]. Genetic mutations in human olfactory receptor gene *TAAR5* may decrease the aversion to the aroma of trimethylamine [99], suggesting that individual differences in sensory perception may influence the acceptance of fermented fish products rich in trimethylamine.

Sulfur-containing compounds like dimethyl sulfide, dimethyl disulfide and dimethyl trisulfide (sulfur, rotten cabbage) may be derived from Strecker degradation of cysteine and methionine [31,39,40]. Dimethyl trisulfide has been positively correlated with the microbial genera *Acinetobacter*, *Citrobacter*, *Streptococcus*, and *Vibrio* [39]. However, some LAB strains, including *Limosilactobacillus fermentum* PCC, *Lactiplantibacillus plantarum* 299v, and *Lactococcus lactis* subsp. *cremoris* DSM 20069 have been found unable to produce volatile nitrogen- and sulfur-containing compounds [40]. The off-odor compounds (dimethyl disulfide, dibutyl phthalate, indole, and isovaleric acid) in fermented mandarin fish can bind to the tyrosine and tryptophan residues of myofibrillar proteins via hydrogen bonds, van der Waals force, or hydrophobic interactions to different extents [100]. The treatment of fermented fish products with hydrophobic bond-disrupting agents (e.g., urea, guanidine hydrochloride, and propylene glycol) could potentially reduce the release of odorous compounds [100]. However, the impact of these chemicals on favorable volatiles remains to be studied.

### 3.3. General Discussion on Characteristic Compounds

During fermentation, the macromolecules (e.g., carbohydrates, lipids, and proteins) in raw materials are gradually broken down into smaller molecules, resulting in the formation of various aroma and taste compounds. The specific flavor profile of fermented products is determined by a combination of factors including the raw materials, microbial community, and fermentation conditions. Even within the same piece of fish muscle, the flavor compounds can vary between the dorsal and ventral sides [39]. Additionally, ingredients like spices can significantly contribute to the enrichment of flavor compounds in the final product [4,30]. Free amino acids, peptides, and volatiles such as aldehydes, ketones, alcohols, and esters greatly contribute to shaping taste and aroma of fermented fish products. The inoculation of specific strains, particularly LAB and *Saccharomyces cerevisiae*, can promote the generation of desirable flavor compounds. Other flavor compounds, such as umami-tasting 5′-nucleotides and nitrogen- and sulfur-containing compounds, also contribute to the unique sensory profiles of these products.

While the studies discussed in this section provide valuable insights into the formation and diversity of flavor compounds in fermented fish products, there are some limitations that need to be addressed. The metabolic pathways and enzymes involved in the formation of key flavor compounds are not fully elucidated, and the interactions between different microorganisms and their impact on the sensory profiles of fermented fish products require further research. To address these limitations, future research should focus on employing advanced analytical techniques such as metabolomics and sensory evaluation methods to provide a more comprehensive understanding of the complex flavor profiles and consumer preferences for these traditional foods. Furthermore, targeted studies using genetic and molecular biology tools can help to identify the specific genes and enzymes responsible for the production of desirable flavor compounds, enabling the development of starter cultures with enhanced flavor-producing capabilities. Finally, optimization of fermentation conditions and the use of appropriate processing techniques such as the removal of off-odor compounds with hydrophobic bond-disrupting agents can further improve the sensory quality of fermented fish products.

## 4. Strategies for Accelerating Fermentation for Fermented Fish Products

Fermentation of fish can be a time-consuming step, and acceleration of this can be an attempt to acquire more economic benefits via boosting production with the same facilities in a shortened period. Accelerating the fermentation process while maintaining product quality and safety is a key challenge for the fermented fish industry. The rate of fermentation is largely determined by the activity of enzymes, particularly lipases and proteases [47,59,82]. Therefore, strategies to enhance the activity of these enzymes, either through the inoculation of microbial starter cultures or the direct addition of exogenous enzymes, can potentially accelerate the fermentation process. Additionally, the manipulation of fermentation conditions such as temperature can also influence the rate of enzymatic reactions and, consequently, the speed of fermentation [34]. In this section, we discuss various strategies for accelerating the fermentation of fish products. The challenges and limitations associated with each approach and areas for future research and development are also highlighted.

### 4.1. Effects of Inoculum

The inoculation of starter cultures is a simple method to shorten the fermentation time of fish products. For example, fermenting carp with a mixed starter culture containing *Pediococcus pentosaceus* 220, *Staphylococcus xylosus* 135, and *Saccharomyces cerevisiae* 22 (1:1:1) at 24 °C can reduce the fermentation time by half compared to spontaneous fermentation (15 days vs. 30 days) [49]. Besides bacteria and yeast, the inoculation of haloarchaea can also accelerate the fermentation of fish. For example, the fermentation of Indian oil sardines with *Halobacterium* sp. S12FS1 had a significantly shorter fermentation time (4 months) compared to spontaneously fermented controls (12 months) [32]. The high proteolytic and lipolytic activity of the inoculated strain was responsible for the rapid breakdown of the fish substrate and the accelerated development of the characteristic flavor and aroma. Thus, the selection of starter cultures with high enzymatic activity is necessary for the successful acceleration of fish fermentation.

### 4.2. Effects of Enzyme Addition

The direct addition of enzymes can accelerate the fermentation process. Previous studies found that the addition of lipase in fermented carp resulted in enrichment of the volatiles and shortened fermentation periods [33,71]. It should be mentioned that the addition of lipase did not significantly change the pH and the bacterial community in spontaneously fermented carp [33], indicating that the acceleration effect was primarily due to the enhanced enzymatic activity. Similarly, the addition of papain (200 or 400 U/g) was able to reduce the fermentation time of *Chouguiyu* at 12 °C from 10 to 7 days [70]. The sensory acceptability of the papain-added fermented fish was comparable with that of the counterpart without additives, suggesting that the acceleration of fermentation did not compromise product quality. Furthermore, the addition of papain elevated the abundance of *Lactiplantibacillus* and *Carnobacterium*, the genera highly correlated with the key volatiles, possibly due to the hydrolysis of proteins promoted the propagation of the LAB strains [70]. In general, the addition of exogenous enzymes is a promising approach for accelerating the fermentation of fish products. However, the successful implementation of this strategy requires a thorough understanding of the specific fermentation ecosystem, the optimization of enzyme type and dosage, and the consideration of regulatory and consumer acceptance issues which required further studies.

### 4.3. Effects of Thermal Treatment

Thermal treatment is another promising strategy for accelerating the fermentation of fish products. In a previous study, conventional heating and ohmic heating for 8 weeks were compared with spontaneously fermented Pacific whiting for fish sauce production [34]. The authors suggested that both thermal treatments accelerated fermentation process. Furthermore, the ohmic-heated samples were found to be advantageous over the conventional heating in terms of appearance and levels of taste-presenting volatiles. The activities of the microorganisms in the ohmic-heated samples were also promoted, leading to the generation of more flavor compounds. However, it is important to note that the histamine content was also higher in the ohmic-heated samples [34]. To mitigate this issue, the inoculation of specific starter cultures may be employed to reduce the formation of biogenic amines in the products. Other thermal treatment methods, such as microwave heating and infrared heating, may also have potential applications in the acceleration of fish fermentation. In addition to the type of heating method, other physical treatments such as electrostatic fermentation [101], moderate electric field [102], and electromagnetic waves [103] may also be explored as potential approaches to accelerate fish fermentation.

### 4.4. General Discussion on Accelerating Fermentation

The strategies discussed in this section offer promising approaches for reducing fermentation time and improving product consistency and predictability. However, there are still some limitations in addressing the challenges associated with accelerating fermentation. While studies have demonstrated the effectiveness of certain microbial strains and enzymes in accelerating fish fermentation [32,33,49,71], their performance may vary depending on the type of fish substrate, the native microbiota, the fermentation conditions, and the target product profile. The use of starter cultures and enzymes may alter the microbial and biochemical dynamics of fermentation, leading to changes in the formation of beneficial or harmful compounds such as flavor precursors, biogenic amines, or Maillard reaction products. Furthermore, while the acceleration of fermentation may improve the production efficiency and consistency of these products, it may also lead to changes in their sensory attributes, nutritional value, and traditional character, which may impact consumer preferences and decisions.

To address these limitations, future research should focus on developing comprehensive screening methods to identify starter cultures with desired enzymatic capabilities while minimizing the risk of biogenic amine formation. This can be achieved by employing gene-sequencing methods to select strains that harbor abundant lipase and protease genes and lack amino acid decarboxylase genes. The direct addition of enzymes can be a viable alternative to accelerate fermentation, as it bypasses the need for relying on the enzymatic capabilities of the inoculated strains and allows for better control over the fermentation process. Different exogenous enzymes (e.g., bacillolysin, ficin, pepsin, subtilisin, and trypsin) should be tested individually or in mixture in future research for the comparison of their capability on accelerated fermentation. However, the optimal type, concentration, and combination of enzymes for specific fish species and fermentation conditions should also be investigated to ensure desired outcomes in terms of fermentation time and product quality. While a higher temperature can facilitate enzymatic action and accelerate fermentation, it also increases the activity of biogenic amine-producing microorganisms [17,42,104]. To mitigate this issue, extra measures must be implemented during thermal treatment to control the growth of these undesirable microorganisms, such as the use of targeted starter cultures or the application of hurdle technology. Sensory evaluation studies should also be conducted to assess the impact of accelerated fermentation on the organoleptic properties and consumer acceptance of the final products.

## 5. Potential Biological Properties of Fermented Fish Products

**Figure 2:**
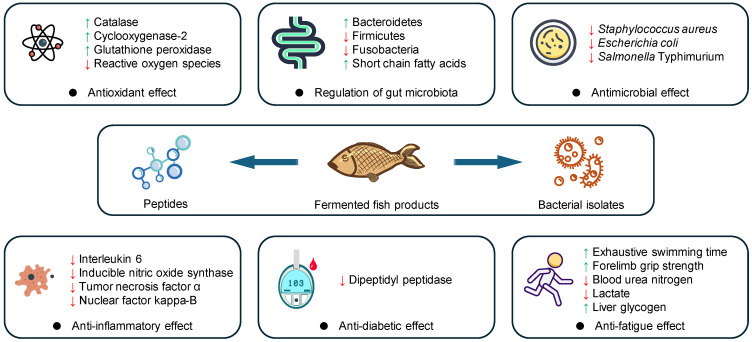
Potential biological effects of fermented fish products. Note: ↑, promotive effects; ↓, inhibitive effect.

The consumption of fermented food products has gained increasing interest among consumers due to their potential health benefits [105]. While a previous review has discussed the biological effects of different fermented fish products and their isolated components [106], there is a need for an up-to-date evaluation of the current evidence and the identification of research gaps and future directions. In this section, we provide an updated overview of the potential biological properties of fermented fish products reported in recent studies, focusing on the scientific evidence, the underlying mechanisms, and the limitations and challenges (Figure 2) (Table 3).

### 5.1. Antioxidant Effect

The antioxidant effects were mainly assessed using chemical assays [35,107,108,109] such as 2,2-diphenyl-1-picrylhydrazyl (DPPH), 2,2′-azino-bis(3-ethylbenzothiazoline-6-sulfonic acid) (ABTS), ferric reducing antioxidant power (FRAP), and cell-based methods [35]. The samples tested in recent studies were in various forms, including whole products, peptide fractions, and microbial isolates. For example, fermented fish products and their isolates exhibited antioxidant effects to different extents [35,107]. Peptides isolated from different Malaysian fermented fish products, such as *budu* and *pekasam*, also demonstrated antioxidant effects [108,109]. Additionally, microbial strain isolates from fermented fish products have also been found to possess antioxidant properties. For instance, a *Lactiplantibacillus plantarum* strain isolated from the Indian fermented fish product *hentak* exhibited concentration-dependent antioxidant activity [110]. These findings suggest that the antioxidant effects of fermented fish products may be attributed to both the fermented matrix and the microbial community involved in the fermentation process. However, these results should be critically reviewed as antioxidant effects deduced from chemical assays have been challenged [111].

**Table 3:** Potential biological properties of fermented fish products.

Products (Region)	Functional Components	Biological Effects and Methods	Major Results	Ref.
*Ka-pi-plaa* (Thailand)	Whole	Antioxidant effect: DPPH, ABTS, FRAP, and metal chelating activity	This product was potential source of natural antioxidants Antioxidant properties increased with the prolongation of fermentation time	[107]
*Budu* (Malaysia)	Peptides (LDDPVFIH and VAAGRTDAGVH)	Antioxidant effect: DPPH, ABTS, and reducing power	Peptide of LDDPVFIH showed higher antioxidant activity The presence of hydrophobic amino acids (Ile and Leu), acidic (Asp) and basic (His) amino acids in the peptide sequences might contribute to the high antioxidant activity	[108]
*Pekasam* (Malaysia)	Peptides (AIPPHPYP and IAEVFLITDPK)	Antioxidant effect: DPPH and ABTS	The IAEVFLITDPK peptide demonstrated higher antioxidant activity than the AIPPHPYP The presence of hydrophobic amino acids (Ile, Ala, and Pro) and basic amino acids (Lys) in the peptide sequences is believed to contribute to the high antioxidant activity	[109]
*Monascus purpureus*-fermented fish bone (Taiwan)	Whole	Antioxidant effect: DPPH, ABTS, and reducing power, hydrogen peroxide-induced Clone-9 cells	↑ Antioxidant activity, ↑ SOD, ↑ CAT, ↑ GPx, ↓ ROS ↑ Mitochondrial membrane potential integrity	[35]
*Hentak* (India)	LAB isolate	Antioxidant effect: DPPH and reducing power	Antioxidant activity increased in a concentration-dependent manner The strain identified as *Lactiplantibacillus plantarum* MK336791	[110]
*Monascus purpureus*-fermented fish bone (Taiwan)	Whole	Anti-inflammation: lipopolysaccharide-induced RAW 264.7 cells	↓ PGE_2_, ↓ IL-6, ↓ TNF-α, ↓ iNOS, ↓ COX-2 ↓ NF-κB via ↑ inhibitor of NF-κB and ↓ p65 & p50	[112]
*Chao* (South Sulawesi, Indonesia)	LAB isolates	Antimicrobial effect: inhibition on *Staphylococcus aureus* and *Escherichia coli*	Functional strain identified as *Lactiplantibacillus plantarum* and *Pediococcus pentosaceus*	[113]
Fermented tilapia (Malaysia)	LAB isolates with various spices (9% turmeric, 6% chili and 9% black pepper)	Antimicrobial effect: inhibition on *Staphylococcus aureus*, *Escherichia coli*, *Salmonella* Typhimurium, *Bacillus cereus*	The highest activity by LAB against *Bacillus cereus* was sample incorporated with black pepper Fermented fish incorporated with chili showed the highest antimicrobial activity against *Staphylococcus aureus*, *Escherichia coli*, and *Salmonella* Typhimurium Higher antimicrobial activity was observed in the presence of spices in comparison to the presence of LAB alone	[44]
Tilapia residue fermented with *Enterococcus faecalis* and *Lactobacillus rhamnosus* (Taiwan)	Whole	Anti-fatigue effect: male senescence-accelerated prone-8 mice	↑ Exhaustive swimming time, ↑ forelimb grip strength ↓ Blood urea nitrogen concentration, ↓ lactate, ↑ liver glycogen	[114]
*Chouguiyu* (China)	Peptides	Dipeptidyl peptidase-IV inhibitory effect: screening assay kit, molecular docking	A total of 125 DPP-IV inhibitory peptides were identified Four novel DPP-IV inhibitory peptides possessing the lowest docking energy were selected, including EPAEAVGDWR, IPHESVDVIK, PDLSKHNNHM, and PFGNTHNNFK	[115]
*Chouguiyu* (China)	Peptides	Dipeptidyl peptidase-IV inhibitory effect: screening assay kit, molecular docking	A total of 278 kinds of amino acid sequences with DPP-IV inhibitory activity were observed A total of 400 dipeptidyl peptidase-IV inhibitors with DPP-IV inhibition structure and high hydrophobicity were identified	[36]
*Monascus purpureus*-fermented grass carp (China)	whole	Regulation of gut microbiota: simulated in vitro fermentation	↓ Abundance of Firmicutes and Fusobacteria ↑ Abundance of Bacteroidetes	[25]
*Surströmming* (Sweden)	Whole	Regulation of gut microbiota: human study	The microbiome of healthy individuals was not affected No global changes in the gut microbiome No members of *Halanaerobium*	[11]

Abbreviations: ABTS, 2,2′-azino-bis(3-ethylbenzothiazoline-6-sulfonic acid); CAT, catalase; COX-2, cyclooxygenase-2; DPPH, 2,2-diphenyl-1-picrylhydrazyl; FRAP, Ferric reducing antioxidant power; GPx, glutathione peroxidase; IL-6, interleukin-6; iNOS, inducible nitric oxide synthase; LAB, lactic acid bacteria; NF-κB, nuclear factor kappa-B; PGE_2_, Prostaglandin E_2_; ROS, reactive oxygen species; TNF-α, tumor necrosis factor-α. Note: ↑, promotive effects; ↓, inhibitive effect.

### 5.2. Anti-Diabetic Effect

Dipeptidyl peptidase-IV (DPP-IV) is a serine protease that plays a key role in the regulation of blood glucose homeostasis by inactivating the incretin hormones such as glucagon-like peptide-1 and glucose-dependent insulinotropic polypeptide. The inhibition of DPP-IV has emerged as a promising therapeutic strategy for the management of type 2 diabetes, as it can prolong the half-life and enhance the biological activity of incretin hormones, leading to improved glucose tolerance and insulin sensitivity [116]. The DPP-IV inhibitory peptides have been found in fermented mandarin fish using in silico methods such as molecular docking and molecular dynamics simulations [36,115]. In the first study, four peptides (EPAEAVGDWR, IPHESVDVIK, PDLSKHNNHM, and PFGNTHNNFK) were found to have the lowest docking energy with the DPP-IV enzyme, suggesting their potential as competitive inhibitors [115]. Mechanistically, their EP-, IPH-, -NHM, and PF- structures can easily interact with the arginine, serine, histidine, and tyrosine residues of the DPP-IV enzyme via hydrogen bonding and salt bridge formation. In the follow-up study, the research group isolated four additional DPP-IV inhibitory peptides (GEKVDFDDIQK, GQKDSYVGDEAQ, KAGARALTDAETAT, and VVDADEMYLKGK) from fermented mandarin fish and confirmed their stability and non-toxicity using in silico tools [36]. While these in silico studies provide valuable insights into the structure–activity relationship of DPP-IV inhibitory peptides from fermented fish products, their actual bioactivity and bioavailability need to be confirmed through in vitro and in vivo experiments. The authors also correlated these peptides with microorganisms such as *Bacillus*, *Kocuria*, *Lactococcus*, *Lysobacter*, and *Peptostreptococcus* [36]. These microorganisms can be isolated and applied as inoculum to enhance the production of DPP-IV inhibitory peptides.

### 5.3. Regulation of Gut Microbiota

The dysbiosis of the gut microbiota has been associated with the pathogenesis of several chronic diseases such as obesity, type 2 diabetes, inflammatory bowel disease, and neurological disorders [117]. Therefore, the modulation of the gut microbiota through dietary interventions, particularly the consumption of fermented foods rich in beneficial microbes and bioactive compounds, can be a promising strategy for promoting health and preventing disease. It was found that the ingestion of the fish fermented with *Monascus purpureus* increased the abundance of Bacteroidetes and decreased the abundance of Firmicutes and Fusobacteria in an in vitro fermentation model, shifting to a healthier gut microbiota profile [25]. The authors also observed an increase in the production of short-chain fatty acids (SCFAs) such as acetic acid, propionic acid, and butyric acid, which are known to have anti-inflammatory and immunomodulatory effects [25]. However, the results obtained from the in vitro assays may not fully reflect the actual biological function in vivo, which need to be further verified by animal and human studies.

In contrast, a clinical study investigated the effect of *surströmming* consumption, and it suggested that the gut microbiota composition of healthy participants was not changed in response to the regular consumption of *surströmming* for up to 60 days [11]. The authors also noted that the genus *Halanaerobium*, as the predominant bacteria in the *surströmming* samples, was not detected in the fecal microbiota of the participants, suggesting the microorganisms in the fermented fish product may not be able to colonize the human gut [11]. A possible reason could be due to the different environment between gastrointestinal tracts and fermented fish (e.g., high salt and low pH), which may limit their survival and growth. Moreover, the study participants were healthy individuals with a presumably balanced gut microbiota, and the impact of *surströmming* consumption on the gut microbiota of people with dysbiosis or specific health conditions remains to be investigated.

### 5.4. Other Biological Functions

In addition to the antioxidant, anti-diabetic, and gut microbiota-modulating effects, fermented fish products have been reported to exhibit other biological functions such as antimicrobial, anti-inflammatory and anti-fatigue activities. The LAB strain isolates from *chao* (Indonesian fermented fish) exhibited antimicrobial effects against common pathogens such as *Staphylococcus aureus* and *Escherichia coli*, as demonstrated by well diffusion assays [113]. The use of spices in the fermentation process may also contribute to the antimicrobial properties of fermented fish products. For example, the fermented fish with 6% chili showed higher inhibitory effects on *Staphylococcus aureus* and *Salmonella* Typhimurium than the products with 9% turmeric or 9% black pepper [44]. Fermented fish products also show an anti-inflammatory effect. For example, *Monascus purpureus*-fermented fish bone down-regulated the levels of pro-inflammatory factors such as interleukin (IL)-6; inducible nitric oxide synthase (iNOS), and tumor necrosis factor (TNF)-α, via the inhibition of nuclear factor kappa-B (NF-κB) [112]. Additionally, fermented fish products have been suggested to have anti-fatigue effects. The tilapia residue fermented with *Enterococcus faecalis* and *Lactobacillus rhamnosus* relieved fatigue in mouse models induced by a weight-loaded swimming test [114].

### 5.5. General Discussion on Potential Biological Properties

While previous review articles have described the potential antioxidant and anti-cancer effects of fermented fish products, these effects were mainly deduced from in vitro studies of isolated compounds such as peptides [106]. The studies discussed in this section also have limitations in providing conclusive evidence for the potential biological properties of fermented fish products. The DPP-IV inhibitory peptides from fermented fish were based on in silico methods [36,115], which may not accurately reflect their actual biological effects in the human body. Similarly, the impact of fermented fish products on gut microbiota was investigated using in vitro fermentation [25] or a small-scale clinical study [11], which may not provide a comprehensive understanding of their effects on the diverse human gut microbiome. Additionally, the impact of fermented fish products on gut microbiota requires confirmation, as the probiotic effects of certain strains (e.g., *Lactobacillus*, *Bifidobacterium*, and *Saccharomyces*) do not necessarily endorse the consumption of fermented fish products as a dietary strategy to acquire probiotics [118]. Many fermented fish products require thermal treatments before consumption [4], which may inactivate live microorganisms and prevent their delivery to the human body. The effects of different cooking methods on the viability of microorganisms within various fermented fish products remain unclear. To address these limitations, future research should focus on conducting well-designed in vivo studies and clinical trials to elucidate the mechanisms of action and potential health benefits of fermented fish products.

Another important aspect that requires attention is the potential health risks associated with the consumption of fermented fish products. The high salinity of these products may pose health risks, as epidemiological studies have evidenced the linkage between high sodium intake and chronic diseases [119,120]. Overemphasizing the health benefits of fermented products may mislead consumers and decrease their awareness of these risks. To address this issue, the development of standardized production methods and quality control measures will be essential to ensure the safety and consistency of fermented fish products. Furthermore, indicating the sodium content in fermented fish products may contribute to rational decision-making regarding purchase and daily consumption for consumers seeking fermented food products that offer health benefits.

## 6. Conclusions and Perspectives

This review has provided an overview of the current state of research on fermented fish products, highlighting the complex interplay between microorganisms, enzymes, and flavor compounds, as well as the potential strategies for improving product quality, safety, and health benefits. The microbial diversity of fermented fish products is influenced by various factors such as the type of raw materials, seasoning ingredients, starter cultures, and fermentation conditions. The formation of flavor compounds including free amino acids, peptides, and volatiles is driven by the action of endogenous and microbial enzymes through complex metabolic pathways. However, the accumulation of biogenic amines poses a significant food safety concern, necessitating the development of effective control measures. Strategies such as the optimization of fermentation conditions, the use of starter cultures, and the application of emerging technologies have shown promise in reducing salt content and accelerating the fermentation process while maintaining product quality. Furthermore, recent studies have suggested potential biological properties of fermented fish products, including antioxidant, anti-diabetic, and gut microbiota-modulating effects, although further research is needed to validate these findings.

Despite the significant progress made in understanding the microbiology, biochemistry, and potential health benefits of fermented fish products, there are still several challenges and opportunities for future research. The development of advanced analytical techniques such as metabolomics and metagenomics can provide a more comprehensive understanding of the complex microbial interactions and metabolic pathways involved in the fermentation process. This knowledge can be leveraged to design tailored starter cultures and optimize fermentation conditions for enhanced flavor, safety, and functionality. Moreover, the application of novel processing technologies such as high-pressure processing and pulsed electric fields may offer new avenues for improving the quality, safety, and shelf-life of fermented fish products while preserving their traditional characteristics. Another area of future research is the exploration of the potential synergistic effects between different starter cultures, enzymes, and processing technologies to maximize the benefits of each approach. Additionally, the development of rapid, non-destructive, and cost-effective methods for monitoring the quality and safety of fermented fish products during storage is crucial for ensuring consumer satisfaction and compliance with regulatory standards. Finally, well-designed in vivo studies and clinical trials are needed to elucidate the mechanisms of action and validate the potential health benefits of fermented fish products, taking into account the safety concerns associated with their consumption. By addressing these challenges and opportunities, researchers and producers can work towards developing fermented fish products that meet the evolving demands of consumers while upholding the rich cultural heritage and diversity of these traditional foods.

## Figures and Tables

**Table 1:** Microorganisms and conditions for various fermented fish products.

Starter Strains	Fish Species	Local Name	Fermentation Conditions	Main Results	Ref.
Solid fermented fish					
*Pediococcus pentosaceus*, *Staphylococcus xylosus*, *Saccharomyces cerevisiae*, and their mixture	Grass carp (*Ctenopharyngodon idellus*)	Not applicable	24 °C for 30 d	↑ Decline of pH and texture softening ↓ Growth of Enterobacteriaceae The mixture of these starter cultures was more conducive to the formation of free amino acids and volatiles than the individual strain	[49]
*Monascus purpureus* Went M 3.439	Grass carp (*Ctenopharyngodon idellus*)	Not applicable	30 °C for 18 h	↑ Free amino acids ↑ Hydrolysis process during in vitro gastric digestion ↓ Ammonia and indole content during in vitro fermentation ↑ Abundance of Parabacteroides in the in vitro fermentation	[25]
*Lactiplantibacillus plantarum*, *Saccharomyces cerevisiae*, and their combination	Grass carp (*Ctenopharyngodon idellus*)	*Suanyu* (China)	30 °C for 15 d	There was no *Staphylococcus aureus*, *Micrococcus*, and *Enterobacteria* in samples with mixed starter cultures *Lactiplantibacillus plantarum* + *Saccharomyces cerevisiae*: umami amino acids increased by 20.56%, sweet amino acids increased by 34.14%, and bitter amino acids decreased by 8.23% Mixed starter cultures were more conducive to the formation of volatiles and pleasant-flavored peptides	[50]
*Lactiplantibacillus plantarum* CGMCC 20032	*Acanthogobius hasta*	*Suanyu* (China)	37 °C for 24 h	Good ability of acid production and acid resistance Inhibition on common pathogens (e.g., *Aeromonas hydrophila*, *Aeromonas veronii*, *Cronobacter sakazakii*, and *Salmonella* Typhimurium)	[51]
Two strains of *Lactiplantibacillus plantarum* and one strain of *Lactococcus lactis*	Grass carp (*Ctenopharyngodon Idella*)	*Yucha* (China)	20 °C for 35 d	↑ Acidification, ↓ biogenic amines, ↑ volatiles The disappearance of 1-methyl-naphthalene might imply mature	[52]
*Latilactobacillus sakei* and *Pediococcus acidilactici*	Tilapia (*Oreochromis mossambicus*)	Not applicable	25 °C for 45 h	Firmicutes ↑ from 69.0% to 81.5% (0–18 h), ↓ to 74.3% (end point) *Latilactobacillus* predominated microbial community after fermentation (18–45 h) with the abundance over 51.5% *Latilactobacillus* contributed the most to the increase of gel strength, texture properties, color, and acidification	[21]
*Lactiplantibacillus plantarum* and Flavourzyme^®^	Grass carp (*Ctenopharyngodon idellus*)	Not applicable	25 °C with 75% humidity for 15 d	Flavourzyme^®^ addition had little effect on the pH at the late fermentation stage ↓ Harmful microbiota (*Enterobacter*, *Staphylococcus aureus*, and *Pseudomonas*) Flavourzyme^®^ addition had no effect on the growth of harmful microbiota Mixture of *Lactiplantibacillus plantarum* and Flavourzyme^®^ was more conducive to lower water activity than *Lactiplantibacillus plantarum* alone ↑ Hydrolysis of protein, ↓ lipid oxidation, ↓ biogenic amines (by 25.11–29.94%)	[53]
Fish paste					
Combination of different lactic bacteria and yeasts	Silver carp (*Hypophthalmichthys molitrix*)	Not applicable	Ambient temperature (25 °C) for 8 weeks	↓ Growth of Enterbacteriaceae and ↓ biogenic amines in all inoculated samples Highest content of sweet-tasting free amino acids was found in group inoculated with the mixture of *Pichia anomala*, *Staphylococcus xylosus*, *Staphylococcus carnosus*, *Pediococcus pentosaceus*, and *Pediococcus acidilactici*	[19]
*Lactiplantibacillus plantarum* 120, *Staphylococcus xylosus* 135, and *Saccharomyces cerevisiae* 31	Common carp (*Cyprinus carpio*)	Not applicable	35 °C for 5 d	↓ Growth of Enterbacteriaceae and ↓ biogenic amines in all inoculated samples Liberation of free fatty acids: *Saccharomyces cerevisiae* 31 > *Staphylococcus xylosus* 135 > *Lactiplantibacillus plantarum* 120 *Lactiplantibacillus plantarum* 120 possessed little or no lipase activity Co-culture with *Lactiplantibacillus plantarum* 120: ↓ growth of *Staphylococcus xylosus* 135, ↑ growth of *Saccharomyces cerevisiae* 31	[54]
*Bacillus velezensis* DZ11	*Hemiculter leucisculus*	Not applicable	25 °C for 40 d	The strain grew well between 20 °C and 30 °C, but the growth ability significantly decreased when the temperature was above 30 °C ↑ Branched chain fatty acids (e.g., as 2-methybutyrate, isovalerate, and 3-hydroxybutyrate), ↑ taste-active amino acids	[55]
*Pediococcus acidilactici* and *Latilactobacillus sakei*	Tilapia (*Oreochromis mossambicus*)	Not applicable	25 °C for 45 h	*Latilactobacillus sakei* was more suitable for tilapia surimi environment than *Pediococcus acidilactici* *Latilactobacillus* and *Lactococcus* had the highest influence on the formation of volatiles	[56]
Fish sauce					
*Limosilactobacillus fermentum* PCC, *Lactiplantibacillus plantarum* 299v, and *Lactococcus lactis* subsp. *cremoris* 20069	Nile tilapia (*Oreochromis niloticus*) fish head	Not applicable	37 °C for 3 d	↑ Aroma compounds (e.g., alcohols and esters), ↓ off-odor notes Samples inoculated with *Limosilactobacillus fermentum* PCC had the most abundant volatiles	[40]
*Latilactobacillus sakei*, *Lactiplantibacillus plantarum*, and *Weissella cibaria*	Mackerel (*Pneumatophorus japonicus*)	Not applicable	30 °C for 5 d	Main volatiles included (E)-2-nonenal, (E,E)-2,4-decadienal, (E)-2-decenal, 1-octen-3-ol, and (E,E)-2,4-nonadienal Higher umami flavor and lower rancid flavor (than commercial fish sauce) *Latilactobacillus sakei* was more conducive to the formation of volatiles than *Lactiplantibacillus plantarum* and *Weissella cibaria*	[57]
*Staphylococcus nepalensis* 5-5	Not applicable	Not applicable	From a fish sauce factory	↓ Biogenic amines (reduction in putrescine, cadaverine, and histamine was 15.74%, 14.18%, and 16.65%, respectively) ↓ Abundance of biogenic amine-positive bacterial genera, ↑ abundance of biogenic amine-negative bacterial genera	[58]
*Halobacterium* sp. S12FS1 in the form of *red yeast rice*	Indian oil sardine (*Sardinella longiceps*)	Not applicable	35 °C for 120 d	Favorable salinity (for the strain): 20–25% (*w*/*v*) salt concentration, cells lysed in the low (5–10% *w*/*v*), and growth delayed at higher salinity (30% *w*/*v*) The best growth (for the strain) at alkaline condition (pH 8), and the growth reduced in alkaline (pH 8.5) and acidic (5–7) conditions ↑ Sensory evolution scores (color, odor, and texture), ↓ biogenic amine	[32]
*Halobacterium salinarum*	Anchovies (*Stolephorus* spp.)	Not applicable	~30−35 °C for up to 180 d	↑ α-Amino group content and total nitrogen content All starters were not biogenic amine formers Dimethyl disulfide (fecal note) lower than that in the commercial fish sauce	[23]
*Aspergillus oryzae* S NPUST-FS-206-A1 in the form of black bean koji	Milkfish (*Chanos chanos*)	Not applicable	30 °C for 6 months	↑ Total soluble nitrogen Histamine was 13.83–16.86 mg/L, lower than the commercial product, and no other biogenic amines detected	[59]

Note: ↑, promotive effects; ↓, inhibitive effect.

## Data Availability

No new data were created or analyzed in this study. Data sharing is not applicable to this article.
